# Analysis of Run-to-Run Variation of Bar-Coded Pyrosequencing for Evaluating Bacterial Community Shifts and Individual Taxa Dynamics

**DOI:** 10.1371/journal.pone.0099414

**Published:** 2014-06-09

**Authors:** Yuan Ge, Joshua P. Schimel, Patricia A. Holden

**Affiliations:** 1 Earth Research Institute, University of California Santa Barbara, Santa Barbara, California, United States of America; 2 Bren School of Environmental Science and Management, University of California Santa Barbara, Santa Barbara, California, United States of America; 3 University of California Center for Environmental Implications of Nanotechnology, University of California Santa Barbara, Santa Barbara, California, United States of America; 4 Department of Ecology, Evolution and Marine Biology, University of California Santa Barbara, Santa Barbara, California, United States of America; American University in Cairo, Egypt

## Abstract

Bar-coded pyrosequencing has been increasingly used due to its fine taxonomic resolution and high throughput. Yet, concerns arise regarding the reproducibility of bar-coded pyrosequencing. We evaluated the run-to-run variation of bar-coded pyrosequencing in detecting bacterial community shifts and taxa dynamics. Our results demonstrate that pyrosequencing is reproducible in evaluating community shifts within a run, but not between runs. Also, the reproducibility of pyrosequencing in detecting individual taxa increased as a function of taxa abundance. Based on our findings: (1) for studies with modest sequencing depth, it is doubtful that data from different pyrosequencing runs can be considered comparable; (2) if multiple pyrosequencing runs are needed to increase the sequencing depth, additional sequencing efforts should be applied to all samples, rather than to selected samples; (3) if pyrosequencing is used for estimating bacterial population dynamics, only the abundant taxa should be considered; (4) for less-abundant taxa, the sequencing depth should be increased to ensure an accurate evaluation of taxon variation trends across samples.

## Introduction

Advances in DNA and RNA sequencing technologies, i.e. 454 pyrosequencing [1], have allowed more sequences to be investigated and thus more taxa within environmental microbial communities to be identified. Although some other next-generation sequencing techniques, e.g., Illumina and Applied Biosystems SOLiD platforms, have higher sequencing throughput than 454 platforms, pyrosequencing remains valuable because of its long read length compared to other next-generation sequencing techniques, which allows potentially more accurate read annotation in ecological applications [2]. Therefore pyrosequencing, after supplanting molecular fingerprinting approaches and Sanger sequencing, remains an important tool in microbial community studies [3], [4].

Although a huge number of sequence reads can be achieved in a single run, the application of pyrosequencing is limited by the high cost of each run. However, a relatively low number of sequences (thousands) per sample are generally sufficient for most research questions in microbial ecology, since aims are usually to explore community shifts and taxa dynamics at phylogenetic levels of genera and above, rather than to describe entire communities at the individual operational taxonomic unit (OTU) level [3], [5]. Moreover, for a given sequencing effort and cost, keeping the number of sequences per sample modest allows for a more robust experimental design, because more samples, replicates, and treatments can be included [6], [7]. To facilitate analyzing large numbers of samples simultaneously, bar-coded pyrosequencing has entered wide use; this allows a single 454 pyrosequencing run (picotitre plate) to process hundreds of samples [8], [9].

A large number of studies have used bar-coded pyrosequencing to explore microbial community shifts and taxa dynamics along various environmental gradients including in pH [10], nitrogen [11], heavy metals [12], elevated CO_2_ [13], warming [14] and drought [15]. In such studies, microbial community differences along the gradient are assumed to exceed the variations due to the methods that are used to analyze the community. Yet, this assumption has not been well tested [16]: the sparse literature has in some cases supported [4] but in others contradicted this assumption [17]. Variations in microbial community analysis can arise at several steps: environmental sample collection, DNA extraction, DNA amplification, amplicon analysis, and data analysis. Minimal methodological variations would be ideal at the stage of amplicon analysis, compared to prior steps, so that intrinsic community differences between samples are not confused with technological limitations.

To quantitatively examine the extent to which run-to-run variation of bar-coded pyrosequencing affects the results of microbial community and population analyses, we used a 454 Genome Sequencer FLX platform to sequence the same bar-coded amplicon library three times: twice on one sequencing plate and the third on a separate half-plate. Our results demonstrate that pyrosequencing is reproducible in evaluating community shifts within a run, but not between runs. Also, the reproducibility of pyrosequencing in detecting individual taxa increased as a function of taxa abundance.

## Materials and Methods

### Soil samples and experiment design

Soil samples were collected from an engineered nanoparticle (ENP) exposure experiment [18], with four experimental replicates per ENP treatment (control, 2 mg g^−1^ soil of nano-TiO_2_, and 0.5 mg g^−1^ soil of nano-ZnO) and sampling time (day 15 and 60). Samples without ENPs were used as controls, and four additional control samples were stored at day 0 for characterization of the baseline soil conditions. A total of 28 soil samples were used to prepare a bar-coded amplicon library that was sequenced three times: twice on one sequencing plate and the third on a separate half-plate. Because the same amplicon library was used, this provides a unique opportunity to separately evaluate pyrosequencing run-to-run and within-run variations, in the absence of other variations typically occurring in microbial community analysis. With this experimental design, we aimed to examine run-to-run variations and reproducibility of bar-coded pyrosequencing when examining bacterial community shifts and taxa dynamics.

### Bar-coded amplicon library preparation

The bar-coded amplicon library was prepared, based on a previously described procedure [19]. In brief, soil DNA was extracted from 0.3 g soil using the Powersoil DNA Isolation Kit (Mo Bio, Carlsbad, USA) according to the manufacturers' instructions. Genes encoding 16S rRNA were PCR-amplified using unique bar-coded primers [8], following the PCR conditions and thermal cycling scheme described previously [19]. For each sample, triplicate PCR runs were pooled to reduce random PCR bias. PCR products were purified using the QIAquick PCR Purification Kit (Qiagen, Valencia, USA), and quantified using the Quant-iT DNA Assay Kit, High Sensitivity (Invitrogen, Eugene, USA). The purified PCR products from each sample were equally pooled by amount, and concentrated to form a bar-coded amplicon library that was used for pyrosequencing [19].

### Pyrosequencing and sequence preprocessing

Pyrosequencing was performed on a 454 Genome Sequencer FLX platform using Titanium chemistry (Roche, Branford, USA). The sequences achieved were preprocessed to remove low-quality sequences and noise using the AmpliconNoise function in QIIME [20], [21]. In brief, the plain-text flowgram file of each technical replicate was quality filtered using the default parameters of AmpliconNoise, truncated to 400 bp, and split into one file per sample based on the unique barcodes. For each split file, PyroNoise scripts, SeqNoise scripts, and Perseus scripts were respectively conducted to remove sequencing errors, single base PCR errors, and PCR chimeras using the default parameters of AmpliconNoise. After AmpliconNoise screening, qualified sequences from all samples and technical replicates were merged into one file. The merged file was used to cluster qualified sequences into universal OTUs (at a 0.03 cutoff) for Bray-Curtis distance-based community analysis. Phylogenetic trees were also clustered using the merged file for Weighted-Unifrac distance-based community analysis, as described previously [19]. To increase the reliability of community comparison among samples with different sequencing depths, we rarefied the qualified sequence counts of all samples to the smallest sequence count (637) among samples through a random subsampling process, and conducted our analyses using the rarefied sample-OTU matrix [19], [22]. To examine bacterial population dynamics, qualified sequences from all samples and technical replicates were also assigned to a set of hierarchical taxa (phylum, class, order, family, and genus) using the program Classifier in the Ribosomal Database Project (http://rdp.cme.msu.edu/classifier/). The pyrosequencing reads have been deposited in the National Center for Biotechnology Information Sequence Read Archive (NCBI SRA) with an Accession number SRP041081.

### Statistical Analysis

Principal coordinates analysis (PCoA) was used to illustrate the effects of different technical replicates of pyrosequencing (both within and between plates) on the estimation of community shift. A Mantel test with 999 permutations was used to test whether different technical replicates of pyrosequencing can reveal similar patterns of bacterial community shifts. Analysis of variance (ANOVA) was used to examine the effects of ENP treatments, pyrosequencing runs, and technical replicates on community dissimilarities (Bray-Curtis and Weighted-Unifrac distances). A Pearson correlation of the relative abundance of each taxon between technical replicates of pyrosequencing was used to estimate the reproducibility of technical replicates in detecting individual taxon variations across samples. Regression analysis was used to quantitatively predict the number of sequences needed to ensure robust reproducibility when using pyrosequencing to estimate individual taxon variations across samples. The “Metastats” function in Mothur [23], [24] was used to determine which taxa were responsible for shifting the samples between pyrosequencing runs and between ENP treatments.

Analyses were conducted using either Mothur [24], QIIME [20], R (http://www.r-project.org/), or SigmaPlot (Systat Software, San Jose, USA).

## Results and Discussion

**Figure 1:**
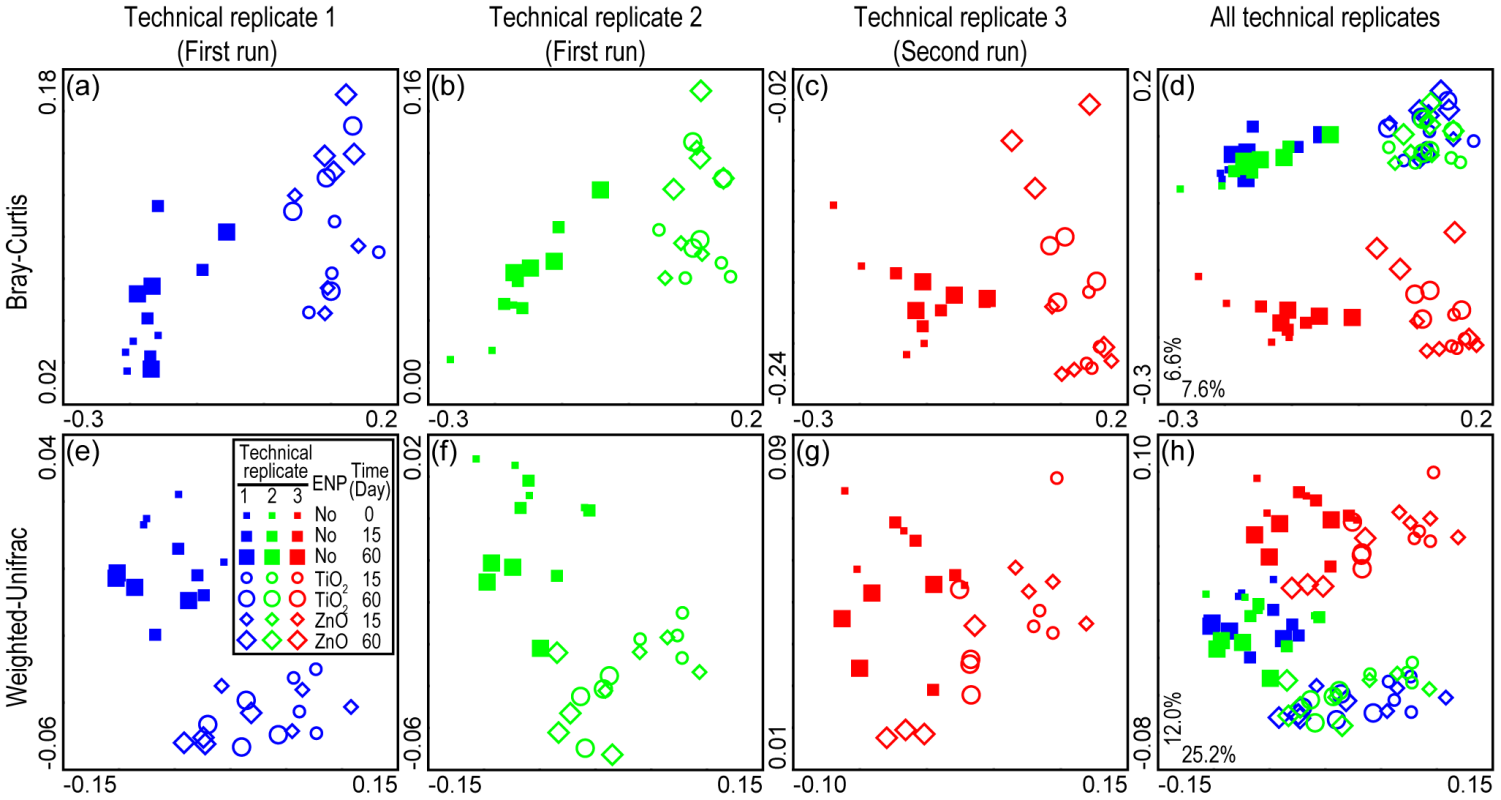
Principal coordinates analysis (PCoA) showing that although each of the three technical replicates was sufficient to reveal community shift in response to nano-TiO_2_ and nano-ZnO (a–c and e–g), bacterial communities derived from technical replicate 3 distinctly separated from the other replicates (d and h). Bacterial community dissimilarity was characterized by Bray-Curtis distance (a–d) and weighted-UniFrac distance (e–h). Technical replicates 1 and 2 were conducted on the same pyrosequencing plate, while technical replicate 3 was sequenced on a separate half-plate.

We first used principal coordinate analysis (PCoA) to illustrate the effects of different technical replicates of pyrosequencing (both within and between plates) on the estimation of community shift. We found that each of three technical replicates was sufficient to reveal bacterial community shifts in response to ENP exposure, as reflected by the distinct separation between controls and nano-TiO_2_ or nano-ZnO treated samples (Fig. 1a–c and e–g). These results indicate that pyrosequencing, whether conducted in the same plate (run) or in a separate plate, is highly reproducible for revealing bacterial community shifts of this magnitude. This was also suggested by the significant pair-wise correlations (*R*>0.6, *P*<0.05 for all combinations) of community dissimilarities derived from three technical replicates (Fig. S1).

**Figure 2:**
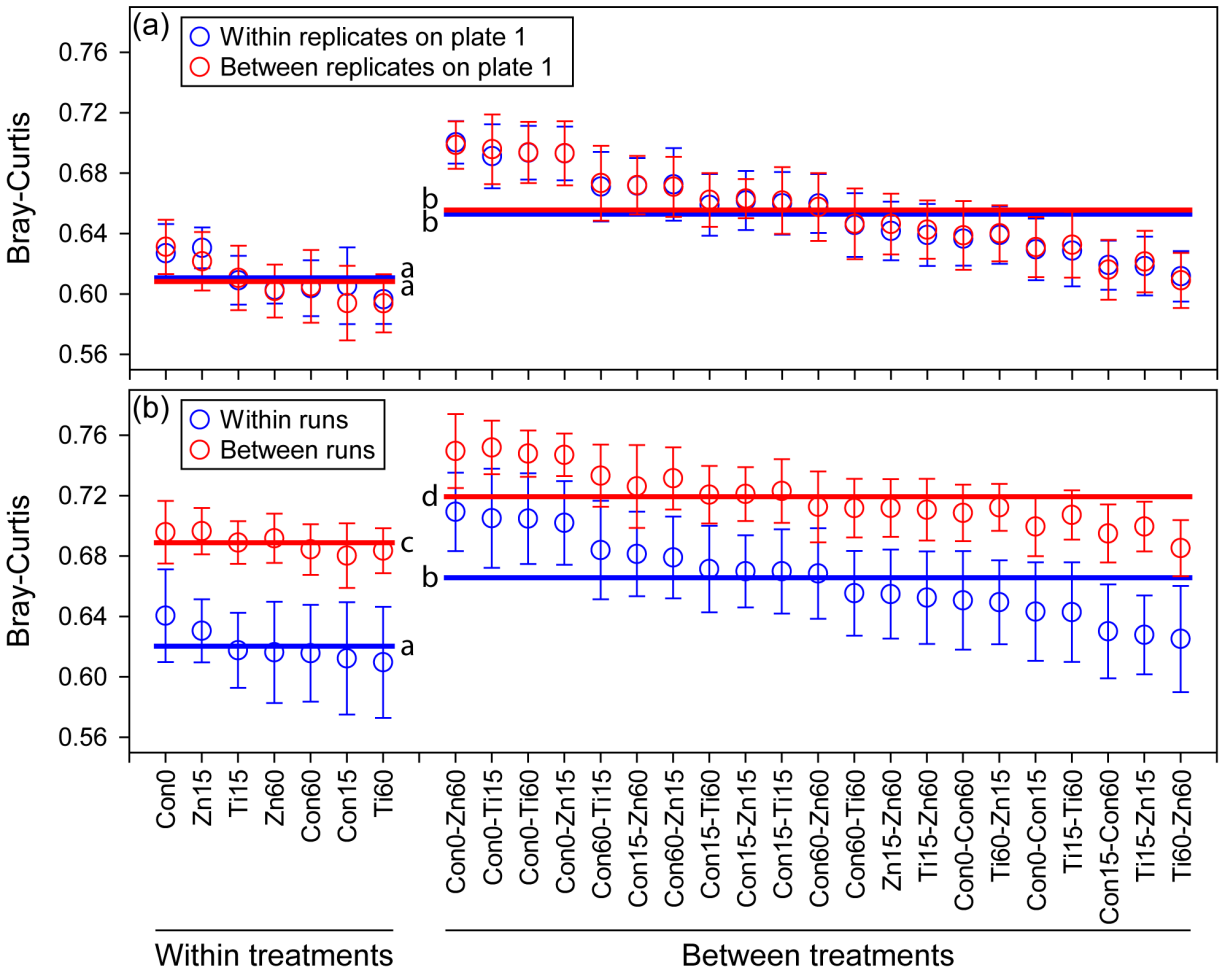
Bray-Curtis distances within and between treatments, technical replicates, and runs, showing that community dissimilarities within and between replicates on the same sequencing plate (technical replicates 1 and 2) were almost identical (a), while community dissimilarities within and between pyrosequencing runs were significantly different (b). The lines represent the mean distances of different groups (within replicates/runs + within treatments, between replicates/runs + within treatments, within replicates/runs + between treatments, between replicates/runs + between treatments). Lines labeled by the same letter do not differ at a *P* value of 0.05. Con, control; Ti, nano-TiO_2_ (2.0 mg g^−1^ soil); Zn, nano-ZnO (0.5 mg g^−1^ soil). Exposure time is indicated by the numerical suffix; e.g., Con15 represents the control at day 15.

However, when we plotted the results of three technical replicates in the same PCoA graph, a distinct run-to-run pyrosequencing effect was observed (Fig. 1d and h). For each sample, the bacterial community resolved from the second pyrosequencing run (technical replicate 3) separated from communities resolved from the first pyrosequencing run (technical replicates 1 and 2), while communities resolved from technical replicates 1 and 2 overlapped each other. This was also suggested by the direct comparisons of community dissimilarities using analysis of variance (ANOVA): for both Bray-Curtis distance (Fig. 2) and Weighted-Unifrac distance (Fig. S2), community dissimilarities within and between replicates on the same sequencing plate (replicates 1 and 2) were almost identical, while community dissimilarities within and between pyrosequencing runs were significantly different (*P*<0.05 for both distances). Notably, community dissimilarities between pyrosequencing runs but within treatments were as high as (for Weighted-Unifrac distance), or significantly higher than (*P*<0.05 for Bray-Curtis distances), community dissimilarities between treatments but within pyrosequencing runs. These results indicate that, compared to the variations within a pyrosequencing run, the run-to-run variation of pyrosequencing in evaluating a community may be relatively high, i.e., high enough to indicate a community shift where in fact one might not exist. A previous study also reported batching effects (i.e. identical samples sequenced at the same sequencing facility and between facilities) that may confuse the interpretation of microbial community data [22].

**Figure 3:**
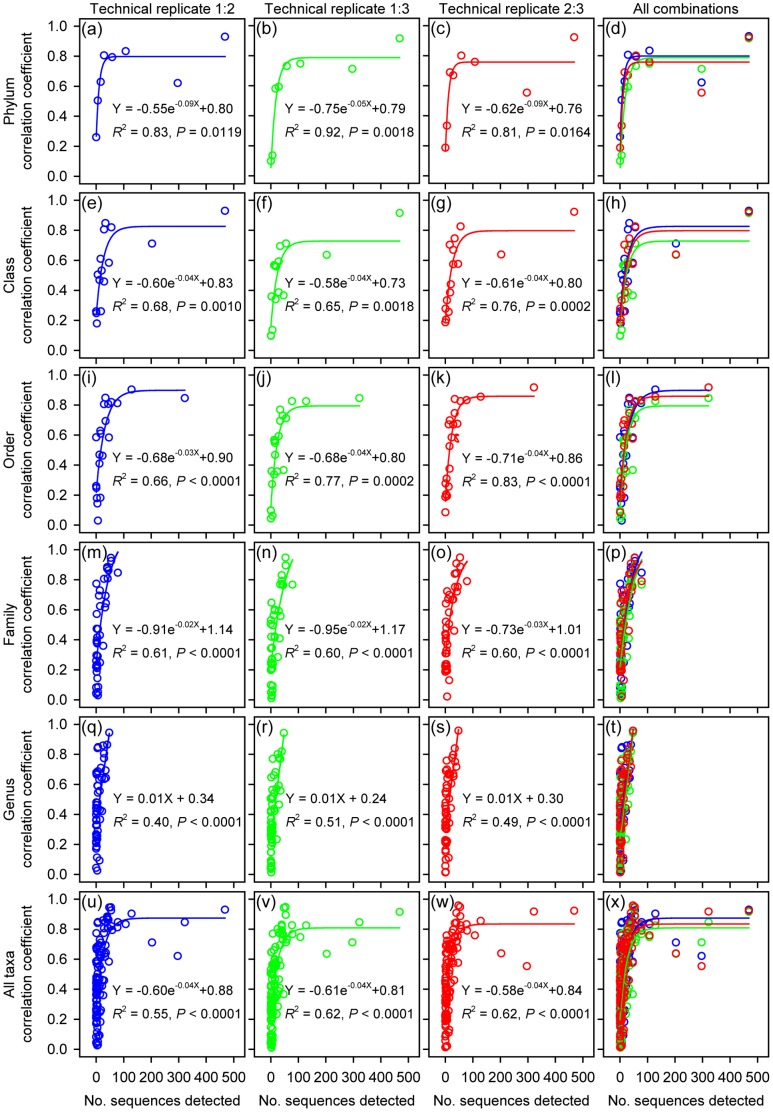
Plots of the reproducibility between technical replicates of taxon relative abundance (calculated as Pearson correlation coefficients) versus the number of detected sequences, showing that the reproducibility of bar-coded pyrosequencing in detecting individual taxon dynamics increased as a function of the detected number of sequences.

We also examined the effects of different technical replicates of pyrosequencing on the estimation of individual taxon variations across samples. To do that, we examined the Pearson correlation of the relative abundance of each taxon between technical replicates of pyrosequencing. Theoretically, a maximum correlation coefficient of 1 should be expected if individual taxon variations across samples could be equally resolved within different technical replicates. We found that, for all pair-wise combinations of technical replicates, the correlation increased as a function of the detected number of sequences (Figs. 3 and S3). These results indicate that it is only for the abundant taxa that variation trends across samples can be resolved repeatedly by different technical replicates. To quantitatively predict the number of sequences needed to ensure robust reproducibility when using pyrosequencing to estimate individual taxon variations across samples, the measure of reproducibility (Pearson correlation coefficient) was exponentially or linearly regressed against the detected number of sequences at different taxonomic levels (Fig. 3). Based on the regression equations, we predicted that, to ensure relatively robust reproducibility, e.g. Pearson correlation coefficient >0.6, the number of sequences detected for a specific taxon should be >23±2 sequences per sample (see also Table S1 for the predicted number of sequences at different taxonomic levels). Until such numbers have been reached, plate-to-plate variation can mask real population variations.

Based on the estimated cutoff, we next used the “Metastats” function in Mothur [23], [24], a non-parametric test, to determine which taxa are responsible for shifting the samples between runs and between treatments. The results showed that, within the 42 classified taxa whose detected number were >23 sequences per sample, 36 taxa were responsible for the observed variation between run 1 and run 2 (*P*<0.05, Table S2), and that 40 taxa were responsible for the observed difference between the control and ENP treatments (*P*<0.05, Table S3). Although we identified the taxa that are responsible for the run-to-run variation, it remains unknown whether this variation is cause by random sampling artifacts during pyrosequencing (emulsion PCR and detection) [17], [25], or by systematic instrument errors. Based on general sampling theory, a recent study has reported that random sampling processes could be an important factor causing high percentages of technical variations for sequencing-based techniques [25].

Our findings have several implications for using bar-coded pyrosequencing to evaluate bacterial community shifts and taxonomic population dynamics. First, although each technical replicate revealed a similar overall pattern of biological community shift, the different pyrosequencing runs were not equivalent in resolving communities at finer resolution (Figs. 1, 2 and S2). Therefore, for those studies with modest sequencing depth, i.e. around 1000 sequences per sample, caution should be taken in interpreting data from different pyrosequencing runs [17], [22]. For example, in this study, bacterial communities in 0-day and 15-day controls overlapped each other. However, if bacterial communities in 0-day controls are evaluated by the first pyrosequencing run (either technical replicate 1 or 2), while bacterial communities in 15-day controls are evaluated by the second pyrosequencing run (technical replicate 3), a community shift between 0-day and 15-day controls would be falsely revealed by PCoA. Furthermore, if multiple pyrosequencing runs are needed in order to increase the sequencing depth, additional sequencing efforts should be applied for all samples, rather than for some selected samples.

Second, our results demonstrated that the reproducibility of pyrosequencing in detecting individual taxon variations across samples increased as a function of the detected number of sequences (Fig. 3). Therefore, to use pyrosequencing to estimate bacterial population dynamics, the taxa should be constrained to abundant taxa, i.e. the detected number should be >23±2 sequences per sample (Table S1), since their variation trends across samples can be analyzed more reproducibly (Pearson correlation coefficient >0.6). On the other hand, for some less-represented taxa, if their variation trends across samples need to be examined, the sequencing depth should be increased to ensure an accurate evaluation [4]; otherwise, a high uncertainty may exist [17].

## Supporting Information

Figure S1 **Significant pair-wise correlations (** ***P*** **<0.05) of community dissimilarities derived from three technical pyrosequencing replicates.** Bacterial community dissimilarity was characterized by Bray-Curtis distance (a–c) and weighted-UniFrac distance (d–f). Technical replicates 1 and 2 were conducted on the same pyrosequencing plate, while technical replicate 3 was on a separate half-plate.
(TIF)

Figure S2 **Weighted-Unifrac distances within and between treatments, technical replicates, and runs, showing that community dissimilarities within and between replicates on the same sequencing plate (technical replicates 1 and 2) were almost identical (a), while community dissimilarities within and between pyrosequencing runs were significantly different (b).** The lines represent the mean distances of different groups (within replicates/runs + within treatments, between replicates/runs + within treatments, within replicates/runs + between treatments, between replicates/runs + between treatments). Lines labeled by the same letter do not differ at a *P* value of 0.05. Con, control; Ti, nano-TiO_2_ (2.0 mg g^−1^ soil); Zn, nano-ZnO (0.5 mg g^−1^ soil). Exposure time is indicated by the numerical suffix; e.g., Con15 represents the control at day 15.
(TIF)

Figure S3 **The Pearson correlation of the relative abundance of each taxon between technical replicates at the phylum level.** Technical replicates 1 and 2 were conducted on the same pyrosequencing plate, while technical replicate 3 was on a separate half-plate. A strong (*R*>0.6) and significant (*P*<0.05) correlation indicates a robust reproducibility of pyrosequencing in detecting individual taxon variations across samples. Each scatterplot matrix shows the results of a specific bacterial phylum, and the detected number of sequences for that phylum is shown in the brackets. Each scatterplot shows the relationship of relative abundance between two technical replicates, which are denoted on the diagonal. The Pearson correlation coefficient for that scatterplot is shown on the corresponding upper right panel, with red color indicating at least *P*<0.05 (*, *P*<0.05; **, *P*<0.01; ***, *P*<0.001). The histogram in the diagonal plot shows the frequency distribution of relative abundance derived from a specific technical replicate.
(TIF)

Table S1 **The predicted number of sequences that is needed to ensure robust reproducibility, e.g. Pearson correlation coefficients of 0.6, 0.7 and 0.8, when using pyrosequencing to estimate individual taxon variations across samples.** Technical replicates 1 and 2 were conducted on the same pyrosequencing plate, while technical replicate 3 was on a separate half-plate.
(PDF)

Table S2 **Taxa responsible for shifting the samples between run 1 and run 2.**
(PDF)

Table S3 **Taxa responsible for shifting the samples between control and ENP treatment.**
(PDF)
