# Supplementary figures and images for: Analysis of Run-to-Run Variation of Bar-Coded Pyrosequencing for Evaluating Bacterial Community Shifts and Individual Taxa Dynamics
Source: PLoS One. 2014 Jun 9;9(6):e99414. doi: 10.1371/journal.pone.0099414 (PMC4049813; doi:10.1371/journal.pone.0099414)

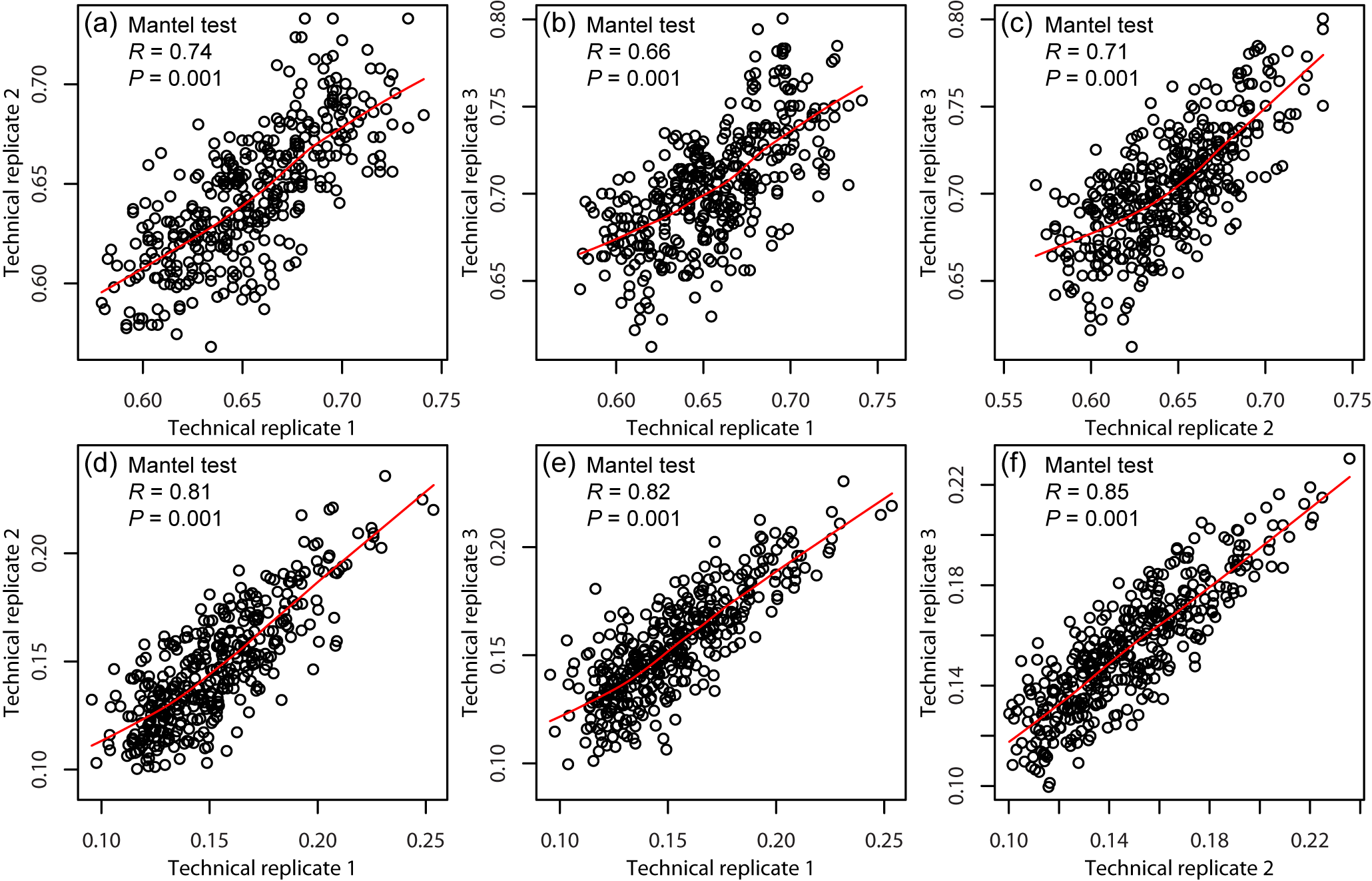

Supplement: Figure S1 — Significant pair-wise correlations ( P <0.05) of community dissimilarities derived from three technical pyrosequencing replicates. Bacterial community dissimilarity was characterized by Bray-Curtis distance (a–c) and weighted-UniFrac distance (d–f). Technical replicates 1 and 2 were conducted on the same pyrosequencing plate, while technical replicate 3 was on a separate half-plate. (TIF) [file pone.0099414.s001.tif]

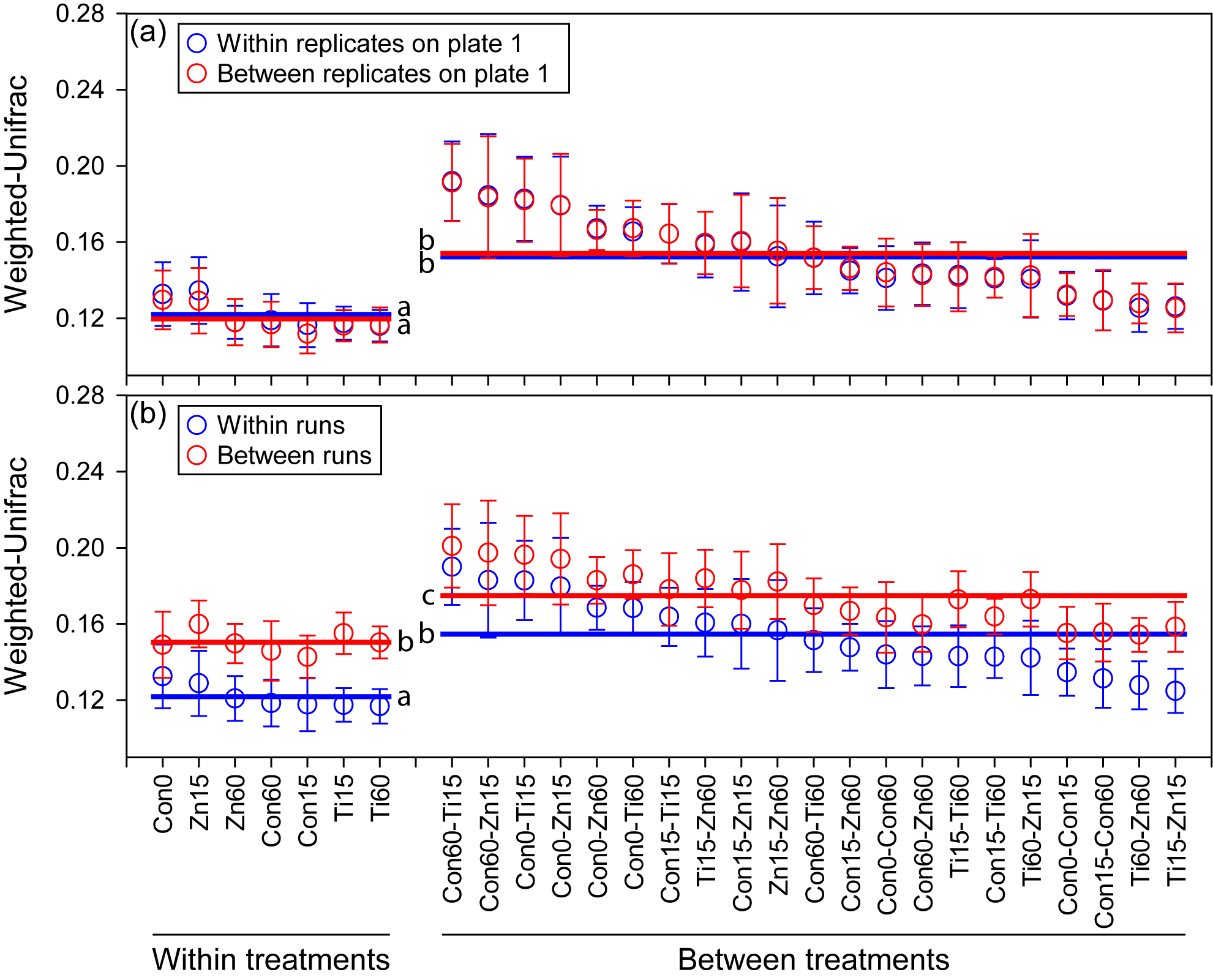

Supplement: Figure S2 — Weighted-Unifrac distances within and between treatments, technical replicates, and runs, showing that community dissimilarities within and between replicates on the same sequencing plate (technical replicates 1 and 2) were almost identical (a), while community dissimilarities within and between pyrosequencing runs were significantly different (b). The lines represent the mean distances of different groups (within replicates/runs + within treatments, between replicates/runs + within treatments, within replicates/runs + between treatments, between replicates/runs + between treatments). Lines labeled by the same letter do not differ at a P value of 0.05. Con, control; Ti, nano-TiO2 (2.0 mg g−1 soil); Zn, nano-ZnO (0.5 mg g−1 soil). Exposure time is indicated by the numerical suffix; e.g., Con15 represents the control at day 15. (TIF) [file pone.0099414.s002.tif]

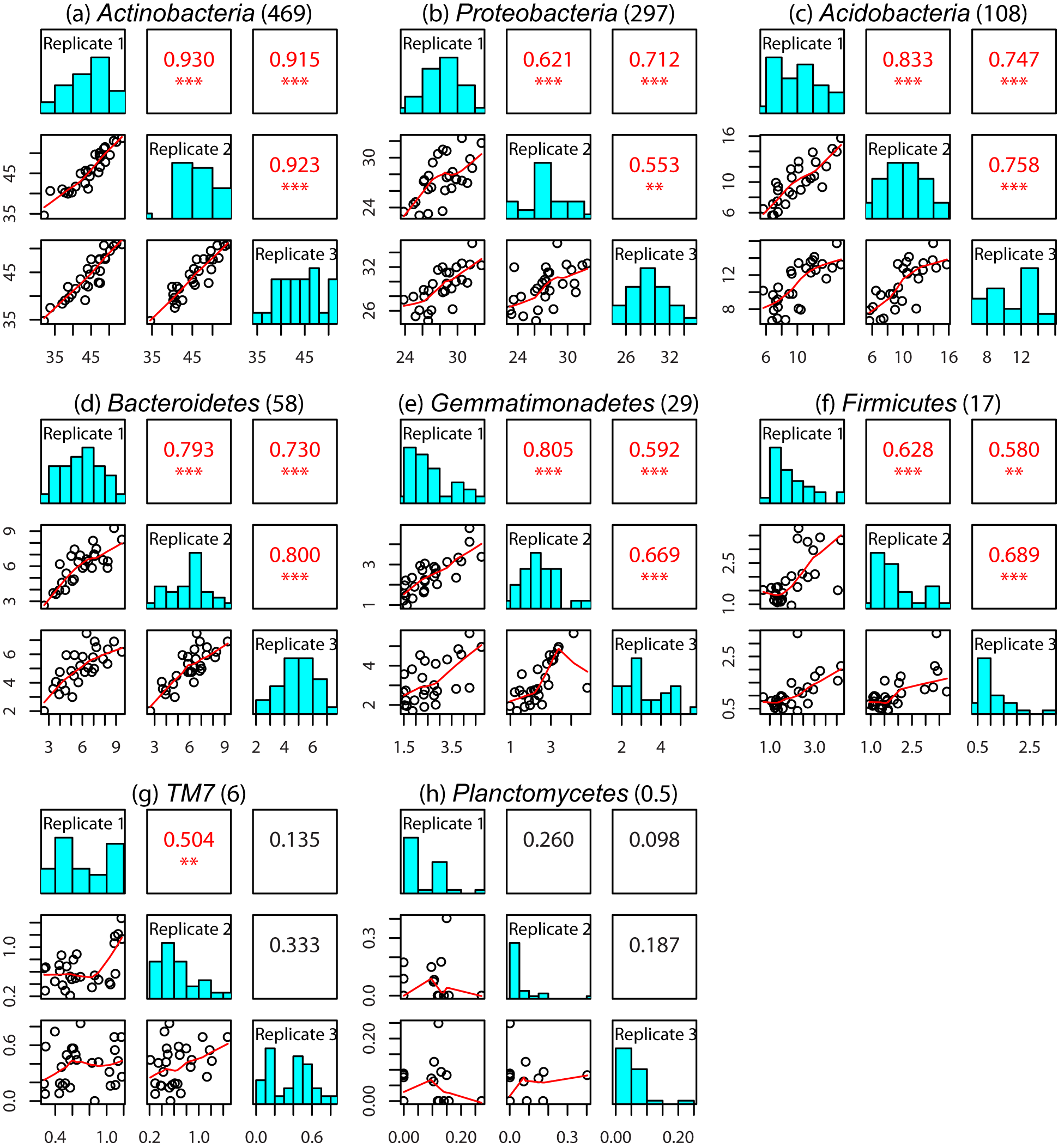

Supplement: Figure S3 — The Pearson correlation of the relative abundance of each taxon between technical replicates at the phylum level. Technical replicates 1 and 2 were conducted on the same pyrosequencing plate, while technical replicate 3 was on a separate half-plate. A strong (R>0.6) and significant (P<0.05) correlation indicates a robust reproducibility of pyrosequencing in detecting individual taxon variations across samples. Each scatterplot matrix shows the results of a specific bacterial phylum, and the detected number of sequences for that phylum is shown in the brackets. Each scatterplot shows the relationship of relative abundance between two technical replicates, which are denoted on the diagonal. The Pearson correlation coefficient for that scatterplot is shown on the corresponding upper right panel, with red color indicating at least P<0.05 (*, P<0.05; **, P<0.01; ***, P<0.001). The histogram in the diagonal plot shows the frequency distribution of relative abundance derived from a specific technical replicate. (TIF) [file pone.0099414.s003.tif]
