# Supplementary material for: Analysis of Run-to-Run Variation of Bar-Coded Pyrosequencing for Evaluating Bacterial Community Shifts and Individual Taxa Dynamics
Source: PLoS One. 2014 Jun 9;9(6):e99414. doi: 10.1371/journal.pone.0099414 (PMC4049813; doi:10.1371/journal.pone.0099414)
Supplement: Table S1 — The predicted number of sequences that is needed to ensure robust reproducibility, e.g. Pearson correlation coefficients of 0.6, 0.7 and 0.8, when using pyrosequencing to estimate individual taxon variations across samples. Technical replicates 1 and 2 were conducted on the same pyrosequencing plate, while technical replicate 3 was on a separate half-plate. (PDF) [file pone.0099414.s004.pdf]

Table S1. The predicted number of sequences that is needed to ensure robust reproducibility, e.g. Pearson correlation coefficients of 0.6, 0.7 and 0.8, when using pyrosequencing to estimate individual taxon variations across samples. Technical replicates 1 and 2 were conducted on the same pyrosequencing plate, while technical replicate 3 was on a separate half-plate.

Taxonomic level	*R*	Technical replicate			Mean	SE	CV (%)
		1:2	1:3	2:3			
Phylum	0.6	11	26	15	18	4	44.1
	0.7	19	40	27	29	6	38.5
	0.8	NA	NA	NA	NA	NA	NA
Class	0.6	26	37	28	30	3	19.2
	0.7	41	74	46	54	10	33.0
	0.8	82	NA	NA	NA	NA	NA
Order	0.6	26	29	23	26	2	12.3
	0.7	39	46	34	40	3	15.3
	0.8	61	NA	56	59	2	5.2
Family	0.6	24	29	22	25	2	14.7
	0.7	33	39	32	35	2	11.3
	0.8	45	53	47	48	2	8.7
Genus	0.6	20	26	22	22	2	13.3
	0.7	28	33	29	30	2	9.2
	0.8	35	40	36	37	1	6.8
All taxa	0.6	21	27	21	23	2	15.8
	0.7	33	43	33	37	3	16.1
	0.8	56	109	64	76	16	37.1
