# Supplementary material for: Analysis of Run-to-Run Variation of Bar-Coded Pyrosequencing for Evaluating Bacterial Community Shifts and Individual Taxa Dynamics
Source: PLoS One. 2014 Jun 9;9(6):e99414. doi: 10.1371/journal.pone.0099414 (PMC4049813; doi:10.1371/journal.pone.0099414)
Supplement: Table S2 — Taxa responsible for shifting the samples between run 1 and run 2. (PDF) [file pone.0099414.s005.pdf]

Table S2. Taxa responsible for shifting the samples between run 1 and run 2.

	Run 1		Run 2		p-value	Taxonomic affiliation
	Mean (%)	SE	Mean (%)	SE		
Taxon004	5.96	0.22	5.03	0.25	0.006	*Bacteroidetes*
Taxon009	2.53	0.12	3.19	0.21	0.006	*Gemmatimonadetes*
Taxon015	27.84	0.33	29.32	0.49	0.011	*Proteobacteria*
Taxon028	3.61	0.23	2.79	0.23	0.017	*Acidobacteria;Acidobacteria_Gp4*
Taxon030	3.83	0.11	5.45	0.26	0.001	*Acidobacteria;Acidobacteria_Gp6*
Taxon054	20.40	0.32	17.94	0.55	0.001	*Proteobacteria;Alphaproteobacteria*
Taxon055	2.01	0.08	4.02	0.18	0.001	*Proteobacteria;Betaproteobacteria*
Taxon048	2.53	0.12	3.19	0.21	0.011	*Gemmatimonadetes;Gemmatimonadetes*
Taxon037	5.68	0.21	4.80	0.24	0.018	*Bacteroidetes;Sphingobacteria*
Taxon120	1.58	0.07	3.27	0.15	0.001	*Proteobacteria;Betaproteobacteria;Burkholderiales*
Taxon106	2.53	0.12	3.19	0.21	0.005	*Gemmatimonadetes;Gemmatimonadetes;Gemmatimonadales*
Taxon075	3.61	0.23	2.79	0.23	0.016	*Acidobacteria;Acidobacteria_Gp4;Gp4*
Taxon077	3.83	0.11	5.45	0.26	0.001	*Acidobacteria;Acidobacteria_Gp6;Gp6*
Taxon115	12.86	0.40	11.44	0.54	0.038	*Proteobacteria;Alphaproteobacteria;Rhizobiales*
Taxon084	2.70	0.14	4.18	0.23	0.001	*Actinobacteria;Actinobacteria;Rubrobacterales*
Taxon085	7.96	0.27	6.50	0.32	0.001	*Actinobacteria;Actinobacteria;Solirubrobacterales*
Taxon090	5.68	0.21	4.80	0.24	0.011	*Bacteroidetes;Sphingobacteria;Sphingobacteriales*
Taxon119	4.77	0.21	3.35	0.17	0.001	*Proteobacteria;Alphaproteobacteria;Sphingomonadales*
Taxon245	8.14	0.32	6.62	0.41	0.008	*Proteobacteria;Alphaproteobacteria;Rhizobiales;Bradyrhizobiaceae*
Taxon201	4.59	0.20	3.91	0.22	0.035	*Bacteroidetes;Sphingobacteria;Sphingobacteriales;Chitinophagaceae*
Taxon234	2.53	0.12	3.19	0.21	0.007	*Gemmatimonadetes;Gemmatimonadetes;Gemmatimonadales;Gemmatimonadaceae*
Taxon170	3.41	0.20	4.36	0.34	0.014	*Actinobacteria;Actinobacteria;Actinomycetales;Geodermatophilaceae*
Taxon174	3.64	0.10	2.13	0.09	0.001	*Actinobacteria;Actinobacteria;Actinomycetales;Microbacteriaceae*

Taxon181	6.21	0.35	3.63	0.34	0.001	*Actinobacteria;Actinobacteria;Actinomycetales;Nocardioidaceae*
Taxon184	2.77	0.11	1.95	0.13	0.001	*Actinobacteria;Actinobacteria;Actinomycetales;Propionibacteriaceae*
Taxon191	2.70	0.14	4.18	0.23	0.001	*Actinobacteria;Actinobacteria;Rubrobacterales;Rubrobacteraceae*
Taxon195	3.78	0.16	2.76	0.17	0.001	*Actinobacteria;Actinobacteria;Solirubrobacterales;Solirubrobacteraceae*
Taxon260	4.61	0.21	3.11	0.17	0.001	*Proteobacteria;Alphaproteobacteria;Sphingomonadales;Sphingomonadaceae*
Taxon187	3.95	0.28	7.07	0.69	0.001	*Actinobacteria;Actinobacteria;Actinomycetales;Streptomycetaceae*
Taxon335	2.42	0.13	3.28	0.25	0.005	*Actinobacteria;Actinobacteria;Actinomycetales;Geodermatophilaceae;Blastococcus*
Taxon531	3.32	0.15	2.12	0.18	0.001	*Proteobacteria;Alphaproteobacteria;Rhizobiales;Bradyrhizobiaceae;Bradyrhizobium*
Taxon504	2.53	0.12	3.19	0.21	0.008	*Gemmatimonadetes;Gemmatimonadetes;Gemmatimonadales;Gemmatimonadaceae;Gemmatimonas*
Taxon396	2.97	0.21	1.45	0.18	0.002	*Actinobacteria;Actinobacteria;Actinomycetales;Nocardioidaceae;Nocardioides*
Taxon425	2.70	0.14	4.18	0.23	0.001	*Actinobacteria;Actinobacteria;Rubrobacterales;Rubrobacteraceae;Rubrobacter*
Taxon429	3.78	0.16	2.76	0.17	0.001	*Actinobacteria;Actinobacteria;Solirubrobacterales;Solirubrobacteraceae;Solirubrobacter*
Taxon416	3.61	0.27	6.13	0.65	0.001	*Actinobacteria;Actinobacteria;Actinomycetales;Streptomycetaceae;Streptomyces*
