# Supplementary material for: Analysis of Run-to-Run Variation of Bar-Coded Pyrosequencing for Evaluating Bacterial Community Shifts and Individual Taxa Dynamics
Source: PLoS One. 2014 Jun 9;9(6):e99414. doi: 10.1371/journal.pone.0099414 (PMC4049813; doi:10.1371/journal.pone.0099414)
Supplement: Table S3 — Taxa responsible for shifting the samples between control and ENP treatment. (PDF) [file pone.0099414.s006.pdf]

Table S3. Taxa responsible for shifting the samples between control and ENP treatment.

	No ENPs		With ENPs		p-value	Taxonomic affiliation
	Mean (%)	SE	Mean (%)	SE		
Taxon001	8.47	0.33	11.72	0.33	0.001	*Acidobacteria*
Taxon002	46.45	0.67	43.48	0.76	0.005	*Actinobacteria*
Taxon004	4.55	0.20	6.48	0.19	0.001	*Bacteroidetes*
Taxon009	2.09	0.10	3.24	0.14	0.001	*Gemmatimonadetes*
Taxon015	29.86	0.38	27.18	0.31	0.001	*Proteobacteria*
Taxon028	2.36	0.20	4.06	0.22	0.001	*Acidobacteria;Acidobacteria_Gp4*
Taxon030	3.77	0.15	4.82	0.20	0.001	*Acidobacteria;Acidobacteria_Gp6*
Taxon033	46.45	0.67	43.48	0.76	0.007	*Actinobacteria;Actinobacteria*
Taxon054	21.63	0.34	18.04	0.32	0.001	*Proteobacteria;Alphaproteobacteria*
Taxon055	2.25	0.17	3.01	0.18	0.003	*Proteobacteria;Betaproteobacteria*
Taxon048	2.09	0.10	3.24	0.14	0.001	*Gemmatimonadetes;Gemmatimonadetes*
Taxon037	4.31	0.19	6.19	0.18	0.001	*Bacteroidetes;Sphingobacteria*
Taxon120	1.82	0.15	2.39	0.15	0.007	*Proteobacteria;Betaproteobacteria;Burkholderiales*
Taxon106	2.09	0.10	3.24	0.14	0.001	*Gemmatimonadetes;Gemmatimonadetes;Gemmatimonadales*
Taxon075	2.36	0.20	4.06	0.22	0.001	*Acidobacteria;Acidobacteria_Gp4;Gp4*
Taxon077	3.77	0.15	4.82	0.20	0.001	*Acidobacteria;Acidobacteria_Gp6;Gp6*
Taxon115	15.21	0.34	10.27	0.20	0.001	*Proteobacteria;Alphaproteobacteria;Rhizobiales*
Taxon084	4.04	0.21	2.56	0.12	0.001	*Actinobacteria;Actinobacteria;Rubrobacterales*
Taxon085	8.87	0.29	6.43	0.22	0.001	*Actinobacteria;Actinobacteria;Solirubrobacterales*
Taxon090	4.31	0.19	6.19	0.18	0.001	*Bacteroidetes;Sphingobacteria;Sphingobacteriales*
Taxon119	3.09	0.13	5.20	0.20	0.001	*Proteobacteria;Alphaproteobacteria;Sphingomonadales*
Taxon245	9.82	0.33	5.99	0.15	0.001	*Proteobacteria;Alphaproteobacteria;Rhizobiales;Bradyrhizobiaceae*
Taxon201	3.32	0.17	5.14	0.17	0.001	*Bacteroidetes;Sphingobacteria;Sphingobacteriales;Chitinophagaceae*

Taxon234	2.09	0.10	3.24	0.14	0.001	*Gemmatimonadetes;Gemmatimonadetes;Gemmatimonadales;Gemmatimonadaceae*
Taxon170	5.28	0.19	2.56	0.11	0.001	*Actinobacteria;Actinobacteria;Actinomycetales;Geodermatophilaceae*
Taxon177	4.97	0.17	2.86	0.13	0.001	*Actinobacteria;Actinobacteria;Actinomycetales;Micromonosporaceae*
Taxon181	3.86	0.27	6.47	0.39	0.001	*Actinobacteria;Actinobacteria;Actinomycetales;Nocardioidaceae*
Taxon184	2.98	0.15	2.13	0.09	0.001	*Actinobacteria;Actinobacteria;Actinomycetales;Propionibacteriaceae*
Taxon191	4.04	0.21	2.56	0.12	0.001	*Actinobacteria;Actinobacteria;Rubrobacterales;Rubrobacteraceae*
Taxon195	4.16	0.18	2.90	0.14	0.001	*Actinobacteria;Actinobacteria;Solirubrobacterales;Solirubrobacteraceae*
Taxon260	2.93	0.13	5.00	0.19	0.001	*Proteobacteria;Alphaproteobacteria;Sphingomonadales;Sphingomonadaceae*
Taxon187	2.35	0.24	6.98	0.34	0.001	*Actinobacteria;Actinobacteria;Actinomycetales;Streptomycetaceae*
Taxon528	5.11	0.20	2.89	0.08	0.001	*Proteobacteria;Alphaproteobacteria;Rhizobiales;Bradyrhizobiaceae;Balneimonas*
Taxon335	3.69	0.16	1.97	0.10	0.001	*Actinobacteria;Actinobacteria;Actinomycetales;Geodermatophilaceae;Blastococcus*
Taxon531	3.74	0.20	2.30	0.10	0.001	*Proteobacteria;Alphaproteobacteria;Rhizobiales;Bradyrhizobiaceae;Bradyrhizobium*
Taxon504	2.09	0.10	3.24	0.14	0.001	*Gemmatimonadetes;Gemmatimonadetes;Gemmatimonadales;Gemmatimonadaceae;Gemmatimonas*
Taxon396	1.55	0.15	3.15	0.23	0.001	*Actinobacteria;Actinobacteria;Actinomycetales;Nocardioidaceae;Nocardioides*
Taxon425	4.04	0.21	2.56	0.12	0.001	*Actinobacteria;Actinobacteria;Rubrobacterales;Rubrobacteraceae;Rubrobacter*
Taxon429	4.16	0.18	2.90	0.14	0.001	*Actinobacteria;Actinobacteria;Solirubrobacterales;Solirubrobacteraceae;Solirubrobacter*
Taxon416	2.04	0.21	6.26	0.32	0.001	*Actinobacteria;Actinobacteria;Actinomycetales;Streptomycetaceae;Streptomyces*
